# 

**Published:** 1900-12

**Authors:** 


### INDEX TO VOLUME XXI.

A.

A Barrier that cannot be swept Away, Gomphosis, 113.
A Bill for the Appointment of Dental Surgeons in the Army, 67.
ABSTRACTS AND TRANSLATIONS:
  Cocaine and its Rational Antidote, 612.
  Errors caused by False Interpretation of Röntgen Rays, 729.
  Gomphosis, a Barrier that cannot be swept Away, 113.
  Micro-Organisms in Dental Caries, 471.
  On the Rôle of Systemic Hyperacidity, 248.
  Syphilitic Locolosis Alveolaris (Pyorrhœa Alveolaris), 664.
  Systemic Hyperacidity and Chemical Abrasion of the Teeth, 316.
  The Antiseptic and Disinfectant Properties of Soap, 171.
  The Necessities of a Medical School, 536.
Academy of Stomatology, 32, 202, 349, 409, 484, 562, 747.
Academy of Stomatology—Resolutions upon the Death of Dr. Bonwill, 64.
Actinomycosis about the Mouth, 522.
A Fatal Case of Hæmophilia, 302.
Allan, Dr. C. F. Is Porcelain Inlay Work Impracticable? 717.
Allan, Dr. George S. Dentistry in its Relation to Medicine, 406.
  Persistently Retarded Temporary Teeth, 509.
Alloys for Filling Purposes, 379.
Alumni of Harvard Dental School, 696.
Alveolar Abscess with Necrosis, A Severe Case of, 123.
Alveolar Fistulas, Headaches from, 705.
Ambler, Henry Lovejoy, M.S., D.D.S., M.D. Facts, Fads, and Fancies about Teeth, 771.
American Academy of Dental Science, 190, 275, 338, 417, 493, 552, 814.
American Medical Association, Section on Stomatology, 367.
A Modified Wire Clasp, 331.
Anæsthetics, The Value of Local, 500.
A New Method of inviting Sleep without Drugs, 462, 638.
Annual and Analytical Cyclopædia of Practical Medicine, 141, 574.
Another Case of Cocaine Poisoning, 706.
Antidote, Cocaine and its Rational, 612.
A Pathological Suggestion, 661.
Apollonia, the "Patron Saint of Dentistry," 1.
A Remarkable Reflex, 640.
A Response to the Toast "Dentistry," 466.
Army, A Bill for the Appointment of Dental Surgeons in the, 67.
Army, Dentists in the, 137.
Arrington, Dr. B. F. Masticating Stress, 647.
  The Etiology of Pyorrhœa Alveolaris discovered so stated, 451.
  What does it mean? 811.
Artificial Caries, 708.
A Severe Case of Alveolar Abscess with Necrosis, 123.
A Suspension Crown, 293.
As we see It, 609.
Autointoxication, Insterstitial Gingivitis due to, 73.

B.

Barker, Dr. C. C. A Response to the Toast "Dentistry," 466.
Barrier that cannot be swept Away, Gomphosis, 113.
Barton, Walter W., D.D.S. Fungoid Pulp, 724.
Beale, Stephen Thomas, M.D., D.D.S., 143.
Beck, Carl. Errors caused by False Interpretation of Röntgen Rays, 729.
BIBLIOGRAPHY:
  Annual and Analytical Cyclopædia of Practical Medicine. By Charles E. de M. Sajous, M.D., 141, 574.
  Dental Metallurgy. By Chas. J. Essig, M.D., D.D.S., 828.

Bibliography:
Discovery of Anæsthesia by Horace Wells, 829.
Facts, Fads, and Fancies about Teeth. By Henry Lovejoy Ambler, M.S., D.D.S., M.D., 771.
Gould's Pocket Pronouncing Dictionary. By George M. Gould, A.M., M.D., 279.
Manual of Pathology. By W. M. Late Coplin, M.D., 825.
Notes on the Treatment of Irregularities in Position of the Teeth. By J. F. Colyer, L.R.C.P., M.R.C.S., 773.
The Practice Builder. By Charles R. Hambly, D.D.S., 364.
Transactions of the National Dental Association, 499.
"Bite," Common-Sense Occlusion, or, 221.
Bite, Is it Possible to jump the? 158.
Boardman, Waldo E., D.M.D. Longevity of Dentists, 376.
Board of Dental Examiners, Commonwealth of Pennsylvania, 218.
Boards, State Dental Examining, and their Questions, 784.
Bogue, E. A., M.D. Consequences of the Extraction of Permanent Teeth, 305.
The Hand the Servant of the Brain, 397.
Bogue, Dr. Frederic L. A Modified Wire Clasp, 331.
Bonwill, Resolutions on the death of Dr., by Academy of Stomatology, 64.
Brain, The Hand the Servant of the, 397.
Brain, Use and Abuse of the, 282.
Briggs, Dr. Edward C. Some of the Nerve Remedies, 659.
Broaches, Swiss Pivot: Their Peculiar Value in preparing Root-Canals, 45.
Burchard, Henry H., M.D., D.D.S., 574.

C.

California State Dental Association, 436.
Caries, Artificial, 708.
Caries, Micro-Organisms in Dental, 471.
Catching, B. H., D.D.S. Gomphosis, a Barrier that cannot be swept Away, 113.
Catching, Dr. Benjamin H., 62.
Central Dental Association of Northern New Jersey, 436.
Changes in the Weather increases Patronage, 707.
Charitable Profession, Is Dentistry a? 359.
Chicago Dental Society, 372, 580.
Clapp, Dwight M., D.M.D. Seventeen Supernumerary Teeth, 645.
Clasp, A Modified Wire, 331.
Clowes, Dr. J. W., 776.
Cocaine and its Rational Antidote, 612.
Cocaine Poisoning, Another case of, 706.
Cocaine, The Extraction of Living Pulps under, 581.
College Man in Dentistry, The, 721.
Colorado State Dental Association, 436.
Colyer, J. F., L.D.S. Notes on the Treatment of Irregularities in Position of the Teeth, 773.
Comments on the Second Application of Arsenic for Devitalization of Pulp, 639.
Common-Sense Occlusion, or "Bite," 221.
Comparative Odontography, 102.
Compressed Air in Operative Dentistry, 162.
Consequences of the Extraction of Permanent Teeth, 305.
Conventions at Old Point Comfort, The, 571.
Coplin, W. M. Late, M.D. Manual of Pathology, 825.
Covering to Pulps, Oxide of Zinc and Eugenol as a, 652.
Criticism, Unjust, 707.
Crown, A Suspension, 293.
Current News:
A Bill for the Appointment of Dental Surgeons in the Army, 67.
American Dental Society of Europe, 850.
American Medical Association, Section on Stomatology, 367.
Board of Dental Examiners, Commonwealth of Pennsylvania, 218, 845.
California State Dental Association, 436.
Central Dental Association of Northern New Jersey, 436, 849.
Chicago Dental Society, 372, 580.
Colorado State Dental Association, 436.
Dental Commissioners of Connecticut, 370, 779.
First District Dental Society of Illinois, 644.

Current News:
Harvard Dental Alumni Association, 641.
Harvard Odontological Society, 418, 643.
Illinois State Dental Society, 291, 371, 579.
Important Notice, 285.
Institute of Dental Pedagogics, 780, 850.
International Dental Congress, 216, 434, 507.
Iowa State Dental Society, 508.
Kentucky State Dental Association, 71, 291, 369.
Lebanon Valley Dental Society, 292.
Massachusetts Board of Registration in Dentistry, 219.
Mississippi Valley Medical Association, 292.
Missouri State Board of Dental Examiners, 219.
Missouri State Dental Society, 372, 507, 643.
National Association of Dental Examiners, 369.
National Association of Dental Faculties, 368, 678.
National Dental Association, 366, 433, 642.
New Dental Society, 641.
New Jersey State Board, Trouble in the, 845.
New York Odontological Society, 72.
New York State Dental Society, 368, 508.
Northeastern Dental Association, 644.
Odontographic Society of Chicago, 580.
Pennsylvania State Dental Society, 371.
Removal, 508.
Resolutions regarding Dr. J. N. Crouse and the Dental Protective Association, 432.
Second District Dental Society, State of New York, 72.
Thirteenth International Medical Congress, 68.
United States Circuit Court, Southern District of New York, 286.
Vermont Board of Dental Examiners, 642.
Wisconsin State Dental Society, 436.

Curtis, G. Lenox, M.D. Cocaine and its Rational Antidote, 612.
Syphilitic Locolosis Alveolaris, 664.
Cushing, Dr. George H., 503.
Cushing, Dr. George H., Resolutions of Respect to, 505.
Cushing, The Death of Dr., 429.

D.

Daly, James H., D.D.S. The Sixth-Year Molar, 49.
Davenport, I. B., M.D. Dentine Plastique du Docteur Klein, 138.
Davis, Edwin E., A. B., D.D.S. A Severe case of Alveolar Abscess with Necrosis, 123.
Death to Mosquitoes, 283.
Death under Nitrous Oxide Gas, 430.
Decision of Judge Townsend, 277.
Dental and Medical School, Union of, 393.
Dental Caries, Micro-Organisms in, 471.
Dental Commissioners of Connecticut, 370, 779.
Dental Degree, What it stands for, 705.
*Dental Digest*, Reply to the, 279.
Dental Education, 458.
Dental Educational Methods, 134.
Dental Educational Requirements, On the Advance in, 431.
Dental Epochs in the Nineteenth Century, 58.
Dental Faculties, National Association of, 363.
Dental Literature of the Past and Present, 208.
Dental Medicine, Education in, 239.
Dental Notes, 235.
Dental Protective Association, 639.
Dental Science, Influences that retard Evolution in, 84.
Dental Service as a Ministry to Life, 402.
Dental Society, Manila, 703.
Dental Stand-Point, Fetor from a, 514.
Dental Surgeons, A Bill for the Appointment of, in the Army, 67.
Dental Work among the Poor: How can it best be accomplished? 597.
Dentine Plastique du Docteur Klein, 138.
Dentist and his Associates, The, 709.
Dentist, The Successful, 654.
Dentistry, a Charitable Profession, Is? 359.

Dentistry, Apollonia the "Patron Saint" of, 1.
"Dentistry," A Response to the Toast, 466.
Dentistry as it appears to the Eye, 245.
Dentistry, Compressed Air in Operative, 162.
Dentistry drifting into Medicine, Is? 495.
Dentistry follows the Flag, 702.
Dentistry in its Relation to Medicine, 406.
Dentistry, Prophylaxis in, 805.
Dentistry, The College Man in, 721.
Dentistry, The Newness of, 389.
Dentists in the Army, 137.
Dentists, Longevity of, 376.
Devitalization of Pulp, Comments on the Second Application of Arsenic for, 639.
Dickerman, Frank R., D.M.D. Effects of Immigration from a Dental View, 384.
Dignity, Professional, 727.
Discovery of Anæsthesia by Horace Wells, 829.
DOMESTIC CORRESPONDENCE:
- Dentine Plastique du Docteur Klein, 138.
- Dr. Clapp's Rejoinder, 831.
- On Methods of applying Silver Nitrate, 778.
- On the Use of Nitrate of Silver, 430.
- Reply to Dr. B. S. Scott, on the Use of "Volasem," 213.
- Reply to the *Dental Digest*, 279.
- Tempering in Glass Tubes, 213.
- The New Method of inducing Sleep without Drugs.—One Hundred Dollar Prize, 638.
- The Value of Local Anæsthetics, 500.
- Wisconsin State Board of Dental Examiners, 64.

Dr. Bogue's Method of treating Sensitive Dentine, 284.
Dr. Bonwill, Resolutions upon the Death of, by Academy of Stomatology, 64.
Dr. Clapp's Rejoinder, 831.
Duties and Responsibilities of the Editor, 426.

E.

Editor, The Duties and Responsibilities of the, 426.
EDITORIAL:
- Charges made by the New Jersey State Board, 824.
- Correction, 824.
- Dental Educational Methods, 134.
- Dental Epochs in the Nineteenth Century, 58.
- Dental Literature of the Past and Present, 208.
- Dentistry follows the Flag, 702.
- Dentists in the Army, 137.
- Dr. Clapp's Reply, 823.
- Is Dentistry a Charitable Profession? 359.
- Is Dentristry drifting into Medicine? 495.
- Manila Dental Society, 703.
- National Association of Dental Faculties, 363.
- Report of Foreign Relations Committee, 704.
- The Conventions at Old Point Comfort, 571.
- The Death of Dr. Cushing, 429.
- The Decision of Judge Townsend, 277.
- The Duties and Responsibilities of the Editor, 426.
- The Galveston Sacrifice, 823.
- The Last Month of the Century, 818.
- The New and the Old, 767.
- The Present and Future of State Boards, 630.
- The Season for Renewed Effort, 698.
- To the Editor of the *Dental Register*, 701.
- What were the Motives? 821.
- What will the Harvest be? 568.

Education, Dental, 458.
Education in Dental Medicine, 239.
Educational Methods, Dental, 134.
Effort, the Season for Renewed, 698.
Eliot, Charles W., L.L.D. The Newness of Dentistry, 389.
Epochs in the Nineteenth Century, Dental, 58.
Errors caused by False Interpretation of Röntgen Rays, 729.
Essig, Dr. Chas. J. Dental Metallurgy, 828.
Etiology of Pyorrhœa Alveolaris discovered so stated, 451.
Evans, W. Warrington, M.D., D.D.S. Common-Sense Occlusion, or "Bite," 221.
Evolution in Dental Science, Influences that retard, 84.
Extirpating Pulps and Subsequent Treatment, Some Methods of, 590.

Extraction of Living Pulps under Cocaine, 581.
Extraction of Permanent Teeth, Consequences of the, 305.

F.

Facing, New Removable, 373.
Facts, Fads, and Fancies about Teeth. By Henry Lovejoy Ambler, M.S., D.D.S., M.D., 771.
Fatal Case of Hæmophilia, A, 302.
Fellows, A. P., D.D.S. Alloys for Filling Purposes, 379.
Fetor from a Dental Stand-Point, 514.
Fillebrown, Thomas A., M.D., D.M.D. A Fatal Case of Hæmophilia, 302.
Filling-Materials, 145.
Fillings, Polishing, 284.
First District Dental Society of Illinois, 644.
Flanagan, Dr. A. J., Dentistry as it appears to the Eye, 245.
Freeman, Dr. S. The Use of Compressed Air in Operative Dentistry, 162.
Fungoid Pulp, 724, 725.
Fungous Growth of the Pulp, 723.

G.

Gingivitis, Interstitial, due to Autointoxication, 73.
Goadby, Kenneth W. Micro-Organisms in Dental Caries, 471.
Gold Scrap, Refining, 285.
Gomphosis, a Barrier that cannot be swept Away, 113.
Gooding, R. L., D.D.S. Fungoid Pulp, 725.
Gordon, Rev. Dr. George A. Dental Service as a Ministry to Life, 402.
Gould, George M., A.M., M.D. Gould's Pocket Pronouncing Dictionary, 279.
Grant, Harry L., D.M.D. The College Man in Dentistry, 721.

H.

Hæmophilia, 295.
Hæmophilia, A Fatal Case of, 302.
Hambly, Charles R., D.D.S. The Practice Builder, 364.
Hand the Servant of the Brain, 397.
Harvard Dental Alumni Association, 641.
Harvard Odontological Society, 418, 643.
Harvest, What will the, be? 568.
Hawes, Dr. Nathaniel Ware, 506.
Headaches from Alveolar Fistulas, 705.
Hopkins, Dr., Reply to, 843.
Howe, Dr. J. Morgan. Notes on Silver Nitrate, 237.
Professional Dignity, 727.
Hyperacidity, On the Rôle of Systemic, 248.
Hypnotic Suggestion in the Practice of Dentistry, 437.

I.

Illinois State Dental Society, 291, 371, 579.
Immigration, Some Effects of, 384.
Important Notice, 285.
Influences that retard Evolution in Dental Science, 84.
Institute of Dental Pedagogics, 780, 850.
International Dental Congress, 216, 434, 507.
International Medical Congress, Thirteenth, 68.
Interstitial Gingivitis due to Autointoxication, 73.
Inviting Sleep without Drugs, A New Method of, 462, 638.
Iowa State Dental Society, 508.
Is Dentistry a Charitable Profession? 359.
Is Dentistry drifting into Medicine? 495.
Is it Possible to jump the Bite? 158.
Is Porcelain Inlay Work Impracticable? 717.

J.

Johnson, C. N., L.D.S., D.D.S. Filling-Materials, 145.

K.

Keen, W. W., M.D. The Necessities of a Medical School, 536.
Kelley, H. A., D.M.D. Dental Work among the Poor: How can it best be accomplished, 597.
Kentucky State Dental Association, 71, 291, 369.
Keyes, Dr. William B., Removal of, 508.
Kimball, Charles Otis, A.B., M.D. The Dentist and his Associates, 709.

L.

Learned, Dr. J. B. A New Method of inviting Sleep without Drugs, 462, 638.
Lebanon Valley Dental Society, 292.
Life, Dental Service as a Ministry to, 402.
Liquid Air as an Appetizer, 284.

Literature of the Past and Present, Dental, 208.
Literature, Some German, of Interest to the Profession, 781.
Local Anæsthesia, The Value of, 500.
Locherty, Dr. James B. Some Methods of extirpating Pulps, and Subsequent Treatment, 590.
Longevity of Dentists, 376.
Luckie, S. B., D.D.S. Oxide of Zinc and Eugenol as a Covering to Pulps, 652.

M.

Manila Dental Society, 703.
Manila Dental Society—Resolutions on the Death of Dr. Theodore Menges and Dr. George H. Cushing, 775.
Martinez, Julio R., D.D.S. Fungous Growth of the Pulp, 723.
Massachusetts Board of Registration in Dentistry, 219.
Massachusetts Dental Society, 49, 123, 358, 434.
Masticating Stress, 647.
Materials, Filling-, 145.
McCall, Charles W., D.D.S., 578.
McClain, John A., D.D.S. A Pathological Suggestion, 661.
Medical Congress, Thirteenth International, 68.
Medical School, The Necessities of a, 536.
Medicine, Dentistry in its Relation to, 406.
Medicine, Is Dentistry drifting into? 495.
Meeker, Dr. Chas. A., Resolutions of Confidence in, 849.
Meeting of American Graduates at Sydney, New South Wales, 215.
Menges, Theodore, B. S., A. M., D.D.S., 501.
Menges, Dr. Theodore, Resolutions of Respect to, 637.
Meriam, Horatio C. A Suspension Crown, 293.
Method of inviting Sleep without Drugs, A New, 462, 638.
Methods of applying Silver Nitrate, 778.
Methods of extirpating Pulps, and Subsequent Treatment, 590.
Michaels, M. On the Rôle of Systemic Hyperacidity, 248.
  Systemic Hyperacidity and Mechanical Abrasion of the Teeth, 316.
Micro-Organisms in Dental Caries, 471.
Miller, W. D., D.D.S., in Reply to Dr. Hopkins, 843.
Mills, Dr. G. Alden. As We see It, 609.
Ministry, Dental Service as a, to Life, 402.
Minot, Dr. C. S. The Union of Dental and Medical School, 393.
Mississippi Valley Medical Association, 292.
Missouri State Board of Dental Examiners, 219.
Missouri State Dental Society, 372, 507, 643.
Mosquitoes, Death to, 283.
Mouth, Actinomycosis about the, 522.

N.

National Association of Dental Examiners, 369.
National Association of Dental Faculties, 363, 368, 678.
National Dental Association, 21, 116, 174, 366, 433, 642.
Neall, E. Henry, D.D.S., 636.
Neall, Walter H., D.D.S. Hypnotic Suggestion in the Practice of Dentistry, 437.
Necessities of a Medical School, The, 536.
Necrosis, A Severe Case of Alveolar Abscess with, 123.
Nerve Remedies, Some of the, 659.
New and Old, The, 767.
New Dental Society, 641.
New Jersey State Dental Society, 756.
New Removable Facing, 373.
New York Institute of Stomatology, The, 39, 181, 256, 329, 417, 541, 615, 669, 738.
New York Odontological Society, 72.
New York State Dental Society, 368, 508.
Newness of Dentistry, 389.
Nineteenth Century, Dental Epochs in the, 58.
Nitrate of Silver, On the Use of, 430.
Nitrous Oxide Gas, Death under, 430.
Northeastern Dental Association, 644.
NOTES AND COMMENTS:
  Amalgam Alloys, 844.
  Another Case of Cocaine Poisoning, 706.
  A Remarkable Reflex, 640.
  A Reply to "An Unjust Criticism," 844.
  Artificial Caries, 708.
  Changes in the Weather increases Patronage, 707.
  Comments on the Second Application of Arsenic for Devitalization of Pulp, 639.

NOTES AND COMMENTS:
Death to Mosquitoes, 283.
Death under Nitrous Oxide Gas, 430.
Dr. Bogue's Method of treating Sensitive Dentine, 284.
Headaches from Alveolar Fistulas, 705.
Liquid Air as an Appetizer, 284.
On the Advance in Dental Educational Requirements, 431.
Perfection in Dental Operations, 431.
Polishing Fillings, 284.
Preparation of Steel for Regulating Appliances, 708.
Refining Gold Scrap, 285.
Remedies for the Office, 432.
Should Formalin be used for Dental Purposes? 706.
Success, 282.
The Dental Protective Association, 639.
Trichloracetic Acid for Fistula from Alveolar Abscess, 707.
Unjust Criticism, 707.
Use and Abuse of the Brain, 282.
What does it profit? 706.
What the Dental Degree stands for, 705.
Notes, Dental, 235.
Notes on Silver Nitrate, 237.
Notes on the Treatment of Irregularities in Position of the Teeth. By J. F. Colyer, L.R.C.P., L.D.S., 773.
Notice, Special, 279.

O.

OBITUARY:
Beale, Stephen Thomas, M.D. D.D.S., 143.
Burchard, Henry H., M.D., D.D.S., 574.
Catching, Dr. Benjamin H., 62.
Clowes, Dr. J. W., 776.
Cushing, Dr. Geo. H., 503, 775.
Hawes, Dr. Nathaniel Ware, 506.
Manila Dental Society—Resolutions on the Death of Dr. Theodore Menges and Dr. George H. Cushing, 775.
McCall, Charles W., D.D.S., 578.
Meeting of American Graduates at Sydney, New South Wales, in Respect to Memory of Dr. Bonwill, 215.
Menges, Theodore, B.S., A.M., D.D.S., 501.

OBITUARY:
Menges, Dr. Theodore, Resolutions of Respect to, 637, 775.
Neall, E. Henry, D.D.S., 636.
Resolutions of Respect to Dr. W. D. Tenison, 366.
Occlusion, Common-Sense, or "Bite," 221.
Odontographic Society of Chicago, 580.
Odontography, Comparative, 102.
Old Point Comfort, The Conventions at, 571.
On the Advance in Dental Educational Requirements, 431.
On the Rôle of Systemic Hyperacidity, 248.
On the Use of Nitrate of Silver, 430.
Operative Dentistry, Compressed Air in, 162.
ORIGINAL COMMUNICATIONS:
Actinomycosis about the Mouth, 522.
A Fatal Case of Hæmophilia, 302.
Alloys for Filling Purposes, 379.
A Modified Wire Clasp, 331.
A New Method of inviting Sleep without Drugs, 462.
A Pathological Suggestion, 661.
Apollonia, the "Patron Saint of Dentistry," 1.
A Response to the Toast "Dentistry," 466.
A Severe Case of Alveolar Abscess with Necrosis, 123.
A Suspension Crown, 293.
As we see It, 609.
Common-Sense Occlusion, or "Bite," 221.
Comparative Odontography, 102.
Consequences of the Extraction of Permanent Teeth, 305.
Dental Education, 458.
Dental Notes, 235.
Dental Service as a Ministry to Life, 402.
Dental Work among the Poor: How can it best be accomplished? 597.
Dentistry as it appears to the Eye, 245.
Dentistry in its Relation to Medicine, 406.
Education in Dental Medicine, 239.
Fetor from a Dental Stand-Point, 514.
Filling-Materials, 145.
Fungoid Pulp, 724, 725.

Original Communications:
Fungous Growth of the Pulp, 723.
Hæmophilia, 295.
Hypnotic Suggestion in the Practice of Dentistry, 437.
Influences that retard Evolution in Dental Science, 84.
Interstitial Gingivitis due to Autointoxication, 73.
Is it Possible to jump the Bite? 158.
Is Porcelain Inlay Work Impracticable? 717.
Longevity of Dentists, 376.
Masticating Stress, 647.
New Removable Facing, 373.
Notes on Silver Nitrate, 237.
Oxide of Zinc and Eugenol as a Covering to Pulps, 652.
Persistently Retarded Temporary Teeth, 509.
Professional Dignity, 727.
Prophylaxis in Dentistry, 805.
Removal of Superior Maxilla and Preservation of Facial Expression, 169.
Sanitary Condition of the Mouth and Teeth, with a Business Method of obtaining the Same, 531.
Seventeen Supernumerary Teeth, 645.
Some Effects of Immigration from a Dental View, 384.
Some German Literature of Interest to the Profession, 781.
Some Methods of extirpating Pulps, and Subsequent Treatment, 590.
Some of the Nerve Remedies, 659.
State Dental Examining Boards and their Questions, 784.
Swiss Pivot Broaches: Their Peculiar Value in preparing Root-Canals, 45.
The College Man in Dentistry, 721.
The Dentist and his Associates, 709.
The Etiology of Pyorrhœa Alveolaris discovered so stated, 451.
The Extraction of Living Pulps from Teeth under the Influence of Cocaine Anæsthesia produced with the Aid of Mechanical Pressure, 581.
The Hand the Servant of the Brain, 397.
The Newness of Dentistry, 389.

Original Communications:
The Sixth-Year Molar, 49.
The Status of our Profession and its Educational Methods in England, as shown in Recent Publications, 3.
The Successful Dentist, 654.
The Union of Dental and Medical School, 393.
The Use of Compressed Air in Operative Dentistry, 162.
What does it mean? 811.
Oxide of Zinc and Eugenol as a Covering to Pulps, 652.

P.

Palmer, Dr. S. B. Influences that retard Evolution in Dental Science, 84.
Palmer, James G. Swiss Pivot Broaches: Their Peculiar Value in Preparing Root-Canals, 45.
Parker, Dr. C. B. Removal of Superior Maxilla and Preservation of Facial Expression, 169.
Pathological Suggestion, A, 661.
"Patron Saint of Dentistry," Apollonia, The, 1.
Peirce, C. N., D.D.S. Apollonia, the "Patron Saint of Dentistry," 1.
Pennsylvania State Dental Society, 371.
Perfection in Dental Operations, 431.
Permanent Teeth, Consequences of the Extraction of, 305.
Persistently Retarded Temporary Teeth, 509.
Polishing Fillings, 284.
Porcelain Inlay Work Impracticable, Is? 717.
Porter, Charles A., M.D. Actinomycosis about the Mouth, 522.
Porter, Charles A., M.D. Hæmophilia, 295.
Potter, Wm. H. Some German Literature of Interest to the Profession, 781.
Practice of Dentistry, Hypnotic Suggestion in the, 437.
Preparation of Steel for Regulating Appliances, 708.
Present and Future of State Boards, 630.
Professional Dignity, 727.
Prophylaxis in Dentistry, 805.
Pulp, Fungoid, 724, 725.
Pulp, Fungous Growth of the, 723.
Pulps, Oxide of Zinc and Eugenol as a Covering to, 652.
Pyorrhœa Alveolaris, Etiology of, discovered, 451.

R.

Rational Antidote to Cocaine, 612.
Raymond, E. H., D.D.S. The Successful Dentist, 654.
Recent Publications, The Status of our Profession and its Educational Methods in England, as shown in, 8.
Reflex, A Remarkable, 640.
Refining Gold Scrap, 285.
Remarkable Reflex, A, 640.
Remedies for the Office, 432.
Remedies, Some of the Nerve, 659.
Removable Facing, New, 373.
Removal, 508.
Removal of Superior Maxilla and Preservation of Facial Expression, 169.
Renewed Effort, The Season for, 698.
Reply to Dr. B. S. Scott on the Use of "Volasem," 213.
Reply to the *Dental Digest*, 279.
Report of Foreign Relations Committee, 704.
REPORTS OF SOCIETY MEETINGS:
  Academy of Stomatology, 32, 202, 349, 409, 484, 562, 747.
  Alumni of Harvard Dental School, 696.
  American Academy of Dental Science, 190, 275, 338, 417, 493, 552, 814.
  Harvard Odontological Society, 418.
  Massachusetts Dental Society, 49, 123, 358, 434.
  National Dental Association, 21, 116, 174, 366.
  New Jersey State Dental Society, 756.
  Report of Foreign Relations Committee of the National Association of Dental Faculties, 678.
  The New York Institute of Stomatology, 39, 181, 256, 329, 417, 541, 615, 669, 738.
  Union Meeting of The New York Institute of Stomatology and the American Academy of Dental Science, 417.
Report of the Foreign Relations Committee of the National Association of Dental Faculties, 678.
Resolutions of Respect to Dr. George H. Cushing, 505.
Resolutions of Respect to Dr. Theodore Menges, 637.
Resolutions of Respect to Dr. W. D. Tenison, 366.
Resolutions regarding Dr. J. N. Crouse and the Dental Protective Association, 432.
Response to the Toast "Dentistry," 466.
Retarded Temporary Teeth, Persistently, 509.
Rollins, William. Dental Notes, 235.
Röntgen Rays, Errors caused by the False Interpretation of, 729.

S.

Sajous, Charles E. de M., M.D. Annual and Analytical Cyclopædia of Practical Medicine, 141, 574.
Sanitary Condition of the Mouth and Teeth, with a Business Method of obtaining the Same, 531.
Second District Dental Society, State of New York, 72.
Sensitive Dentine, Dr. Bogue's Method of treating, 284.
Seventeen Supernumerary Teeth, 645.
Should Formalin be used for Dental Purposes? 706.
Silver Nitrate, Methods of applying, 778.
Silver Nitrate, Notes on, 237.
Sixth-Year Molar, 49.
Smith, B. Holly, M.D. Fetor from a Dental Stand-Point, 514.
Smith, D. D., D.D.S. Prophylaxis in Dentistry, 805.
Soap, The Antiseptic and Disinfectant Properties of, 171.
Some Effects of Immigration from a Dental View, 384.
Some German Literature of Interest to the Profession, 781.
Some Methods of extirpating Pulps, and Subsequent Treatment, 590.
Some of the Nerve Remedies, 659.
Special Notice, 279.
State Boards, The Present and Future of, 630.
State Dental Examining Boards and their Questions, 784.
Status of our Profession and its Educational Methods in England, as shown in Recent Publications, 3.
Stress, Masticating, 647.
Success, 282.
Successful Dentist, The, 654.
Suggestion, A Pathological, 661.
Supernumerary Teeth, Seventeen, 645.
Suspension Crown, A, 293.
Swiss Pivot Broaches: Their Peculiar Value in preparing Root-Canals, 45.
Syphilitic Locolosis Alveolaris (Pyorrhœa Alveolaris), 664.
Systemic Hyperacidity and Mechanical Abrasion of the Teeth, 316.

Systemic Hyperacidity, On the Rôle of, 248.

T.

Talbot, Eugene S., M.D., D.D.S. Dental Education, 458.
Interstitial Gingivitis, due to Autointoxication, 73.
Is it Possible to jump the Bite? 158.
Taylor, Dr. Levi C. Sanitary Condition of the Mouth and Teeth, with a Business Method of obtaining the Same, 531.
Teeth, Consequences of the Extraction of Permanent, 305.
Teeth, Persistently Retarded Temporary, 509.
Teeth, Seventeen Supernumerary, 645.
Tempering in Glass Tubes, 213.
Temporary Teeth, Persistently Retarded, 509.
Tenison, Dr. W. D., Resolutions of Respect to, 366.
The Antiseptic and Disinfectant Properties of Soap, 171.
The College Man in Dentistry, 721.
The Conventions at Old Point Comfort, 571.
The Death of Dr. Cushing, 429.
The Decision of Judge Townsend, 277.
The Dental Protective Association, 639.
The Dentist and his Associates, 709.
The Duties and Responsibilities of the Editor, 426.
The Etiology of Pyorrhœa Alveolaris discovered so stated, 451.
The Extraction of Living Pulps from Teeth under the Influence of Cocaine Anæsthesia produced with the Aid of Mechanical Pressure, 581.
The Hand the Servant of the Brain, 397.
The Last Month of the Century, 818.
The Necessities of a Medical School, 536.
The New and the Old, 767.
The Newness of Dentistry, 389.
The "Patron Saint of Dentistry," Apollonia, 1.
The Practice Builder, 364.
The Present and Future of State Boards, 630.
The Season for Renewed Effort, 698.
The Sixth-Year Molar, 49.
The Status of our Profession and its Educational Methods in England, as shown in Recent Publications, 3.
The Successful Dentist, 654.
The Union of Dental and Medical School, 393.
The Use of Compressed Air in Operative Dentistry, 162.
The Value of Local Anæsthetics, 500.
Thirteenth International Medical Congress, 68.
Thompson, A. H., D.D.S. Comparative Odontography, 102.
Toast "Dentistry," A Response to the, 466.
To the Editor of the *Dental Register*, 701.
Townsend, Decision of Judge, 277.
Transactions of the National Dental Association, 499.
Trichloracetic Acid for Fistula from Alveolar Abscess, 707.
Trueman, William H., D.D.S. The Status of our Profession and its Educational Methods in England, as shown in Recent Publications, 3.
State Dental Examining Boards and their Questions, 784.

U.

Union Meeting of The New York Institute of Stomatology and the American Academy of Dental Science, 417.
Union of Dental and Medical School, 393.
United States Circuit Court, Southern District of New York, 286.
Unjust Criticism, 707.
Use and Abuse of the Brain, 282.

V.

Value of Local Anæsthetics, The, 500.
Vermont Board of Dental Examiners, 642.

W.

What does it mean? 811.
What does it profit? 706.
What the Dental Degree stands for, 705.
What will the Harvest be? 568.
Whitney, Cephas, D.D.S. New Removable Facing, 373.
Williams, Harold, M.D. Education in Dental Medicine, 239.
Wire Clasp, A Modified, 331.
Wisconsin State Board of Dental Examiners, 64.
Wisconsin State Dental Society, 436.

Z.

Zerfing, Wilson, D.D.S. The Extraction of Living Pulps under Cocaine, etc., 581.

